# Klotho enhances FoxO3-mediated manganese superoxide dismutase expression by negatively regulating PI3K/AKT pathway during tacrolimus-induced oxidative stress

**DOI:** 10.1038/cddis.2017.365

**Published:** 2017-08-03

**Authors:** Sun Woo Lim, Long Jin, Kang Luo, Jian Jin, Yoo Jin Shin, Sung Yi Hong, Chul Woo Yang

**Affiliations:** 1 Transplant Research Center, Seoul St. Mary's Hospital, College of Medicine, The Catholic University of Korea, Seoul, Korea; 2 Institute for Convergent Research Consortium for Immunologic Disease, Seoul St. Mary's Hospital, College of Medicine, The Catholic University of Korea, Seoul, Korea; 3 Division of Nephrology, Department of Internal Medicine, Seoul St. Mary’s Hospital, College of Medicine, The Catholic University of Korea, Seoul, Korea

## Abstract

Mammalian members of the forkhead box protein O (FoxO) class of transcription factors are implicated in the regulation of oxidative stress, and FoxO proteins are negatively regulated by the phosphatidylinositol 3-kinase (PI3K)–AKT signaling pathway. We examined the effect of Klotho on the PI3K/AKT pathway and manganese superoxide dismutase (MnSOD) during tacrolimus (Tac)-induced oxidative stress. Klotho-treated mice showed decreased Tac-induced oxidative stress accompanied by functional and histological improvements. Klotho inhibited the PI3K/AKT-mediated phosphorylation of FoxO3a and enhanced FoxO3a binding to the MnSOD promoter. Klotho increased MnSOD mRNA and protein expression in mitochondria. In addition, Klotho reduced Tac-induced mitochondrial dysfunction and decreased mitochondrial reactive oxygen species production, and these effects were enhanced by blocking PI3K activity with LY294002. Collectively, our data showed that Klotho protects Tac-induced oxidative stress by negatively regulating the PI3K/AKT pathway and subsequently enhancing FoxO3a-mediated MnSOD expression.

Klotho, an anti-aging protein, is predominantly expressed in the brain and kidneys,^1^ it extends the mouse lifespan by 20–30%,^2^ and Klotho-deficient mice show multiple age-related phenotypes and experience premature death.^1, 3^ Importantly, recent data showed an association between human longevity and a functional variant of Klotho.^4^ A recent report showed that Klotho overexpression in mice extended the lifespan by repressing of insulin or insulin-like growth factor-1 signaling, an evolutionarily conserved mechanism for lifespan extension.^2^ Yamamoto *et al.*^5^ reported that Klotho-induced expression of the manganese superoxide dismutase (MnSOD) protein, a mitochondrial antioxidant enzyme that detoxifies superoxides, in COS, HELA, and CHO cells and that the anti-oxidative effect of Klotho potentially contributes to its anti-aging properties. Findings related to the roles of Klotho in renal function have shown that Klotho-gene expression levels are significantly decreased in the kidneys of patients with chronic renal failure^6^ and ischemia–reperfusion injury.^7^ Klotho overexpression can preserve renal function by suppressing the development of renal failure^7^ and glomerulonephritis-induced cellular senescence.^8^ However, the regulatory mechanism of Klotho in renal disease remains poorly understood.

Forkhead box protein O (FoxO) transcription factors regulate various downstream target genes, including those involved in cellular differentiation, growth, survival, the cell cycle, glucose and lipid metabolism, stress, and reactive oxygen species (ROS) detoxification.^9^ The phosphatidylinositol 3-kinase (PI3K)-Akt serine-threonine kinase (AKT) signaling pathway regulates FoxO through phosphorylation. The AKT-mediated phosphorylation of FoxO inhibits FoxO activity by promoting its interaction with 14-3-3 proteins and nuclear exportation, and also by inducing its proteasomal degradation.^10^ FoxO3a can upregulate MnSOD expression.^2, 5, 11^ Thus, FoxO3a functions as a negative regulator of mitochondrial ROS production,^12^ and thereby closely associates with resistance to oxidative stress.

Calcineurin inhibitor (CNI)-based regimens are the most popular immunosuppressive drugs used for solid organ transplantation, and 2 CNIs (cyclosporine (CsA) and tacrolimus (Tac)) are available in clinical practice.^13^ In T cells, CsA bind to cyclophilin and Tac binds to FKBP12.^14, 15, 16, 17^ CsA-cyclophilin and Tac-FKBP12 complexes can bind to calcineurin, resulting in inhibition of the phosphatase activity of calcineurin. This inhibition then impairs translocation of the nuclear factor of activated T cells,^18, 19, 20^ which regulates IL-2 transcription and thus T-cell activation.^21, 22, 23^ Despite the specific inhibition of T-cell activation, long-term treatment with CNIs causes serious adverse effects, and nephrotoxicity is a major issue in solid organ transplantation. The pathogenesis of CNI-induced nephrotoxicity is still undetermined, but oxidative stress caused by ROS is regarded as a common pathway of CNI-induced renal injury.^24^

We previously demonstrated that CNI-induced nephropathy was improved by combined treatment with an anti-oxidative agent such as statin, angiotensin II blockade, or N-acetylcysteine.^25, 26, 27, 28^ We also found a causal relationship between Klotho expression and the renoprotective effects of antioxidant agents. Using Klotho +/− mice, we found that Klotho deficiency renders the kidney more susceptible to Tac-induced injury, which was closely associated with aggravated Tac-induced oxidative stress and reduced MnSOD and nuclear FoxO expression.^29^ The current study focused on whether Tac-induced oxidative stress could be inhibited by exogenous treatment with the Klotho protein, which can reduces Tac-induced oxidative stress by FoxO3a-mediated MnSOD expression via negative regulating of the PI3K/AKT pathway.

## Results

### Klotho ameliorates Tac-induced nephrotoxicity

**Figure 1:**
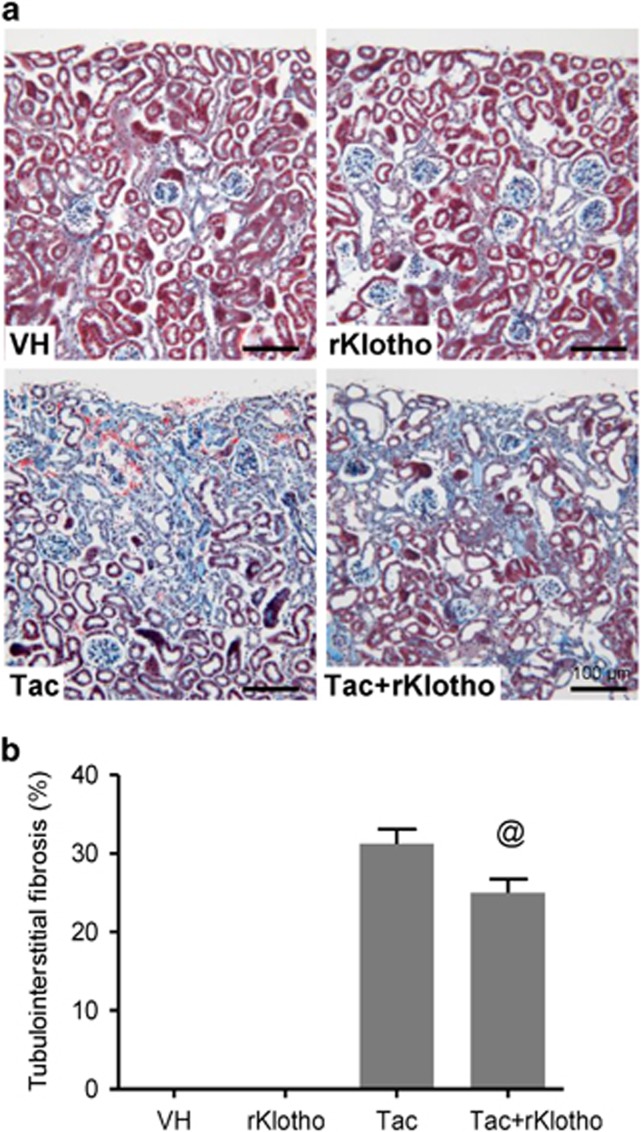
Effect of rKlotho administration on Tac-induced tubulointerstitial fibrosis in an experimental mouse model. (**a** and **b**) Histological analysis in the renal cortex in mice treated with Tac for 4 weeks showed striped tubulointerstitial fibrosis, mononuclear cell infiltration, and tubular atrophy. rKlotho treatment significantly reduced these damages compared with Tac treatment. Scale bar=100 *μ*m. The data are presented as the mean±S.E. ^@^*P*<0.05 versus the Tac group

Table 1 shows changes in the functional parameters in the experimental group after Tac and recombinant Klotho (rKlotho) treatment for 4 weeks. The Tac+rKlotho group showed a lower level of water intake, urine volume, and serum creatinine (Scr) than did the Tac group. Immunoreactivity against renal kidney injury marker-1 (KIM-1) and tubulointerstitial fibrosis (Figure 1) in tissue sections was reduced by Tac treatment in the Tac+rKlotho group.

### Effect of Klotho administration on Tac-induced oxidative stress and apoptosis in mice

**Figure 2:**
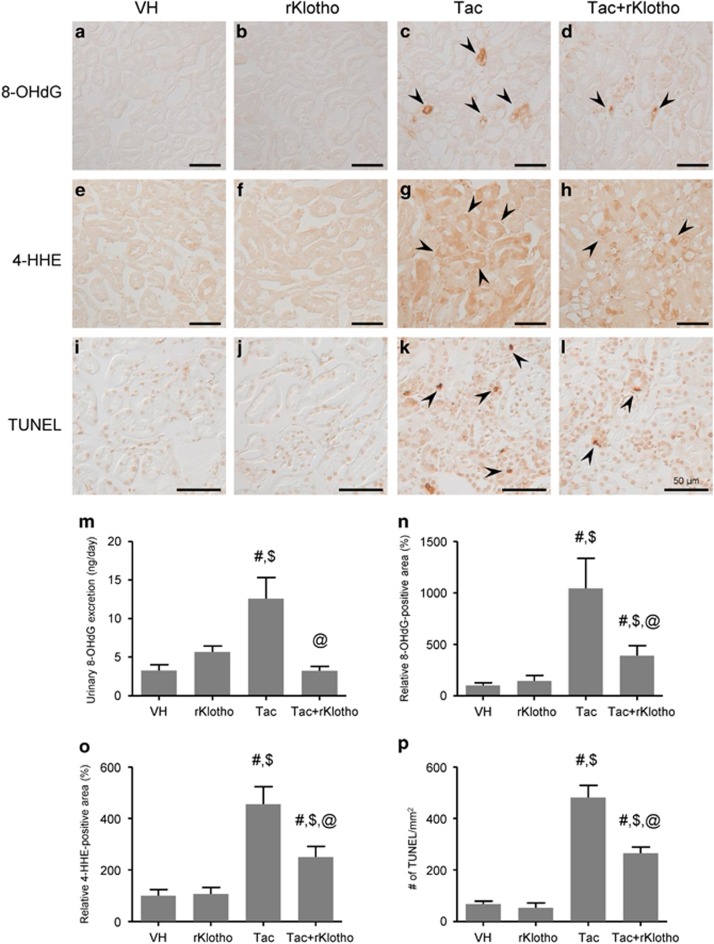
Effect of rKlotho administration on Tac-induced oxidative stress and apoptosis in an experimental mouse model. Representative images and quantification of immunohistochemistry for 8-OHdG (**a**–**d** and **n**) and 4-HHE (**e**–**h** and **o**) and TUNEL assays (**i**–**l** and **p**), using tissue sections from mouse kidneys. High immunoreactivity was significantly decreased by the co-administration of rKlotho. (**m**) Urinary 8-OHdG excretion per day. Tac-induced 8-OHdG excretion was lowered by rKlotho co-administration. The arrows indicate 8-OHdG, 4-HHE, and TUNEL-positives. Scale bar=50 *μ*m. The data are presented as the mean±S.E. ^#^*P*<0.05 versus the VH group; ^$^*P*<0.05 versus the rKlotho groups; ^@^*P*<0.05 versus the Tac group

Figure 2 shows immunohistochemical staining results for 8-hydroxy-2′deoxyguanosine (8-OHdG; Figures 2a–d) and 4-hydroxy-hexenal (4-HHE; Figures 2e–h), and 24-h urinary excretion of 8-OHdG (Figure 2m), a marker of oxidative DNA or lipid damage. Immunoreactivity against 8-OHdG and 4-HHE, and the urine 8-OHdG concentration markedly increased in the Tac group, which was reversed by rKlotho treatment. We also evaluated whether Klotho protects against Tac-induced apoptosis, which is an important cell death mechanism in Tac-induced nephrotoxicity. The number of terminal deoxynucleotidyl transferase dUTP nick end labeling (TUNEL)-positive cells in tissue sections was significantly higher in the Tac group versus the vehicle (VH) group, and this was reduced by the addition of rKlotho (Figures 2i–l, p).

### Effect of Klotho administration on Tac-induced mitochondrial ultrastructure and MnSOD expression in mice

**Figure 3:**
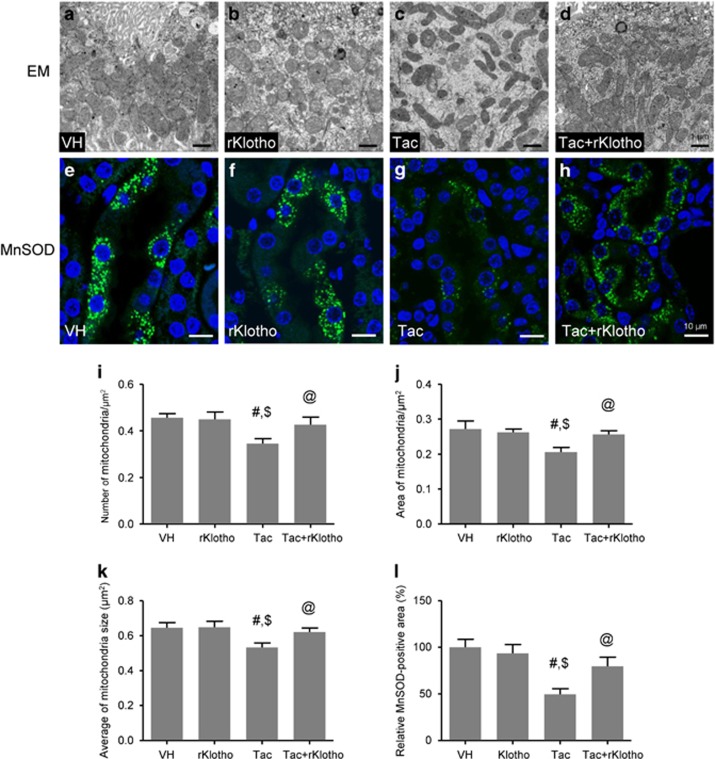
Effect of rKlotho administration on Tac-induced mitochondrial ultrastructure and MnSOD expression in an experimental mouse model. Representative transmission electron microscopy images of the mitochondrial ultrastructure in proximal tubules (**a**–**d**) and its quantification in a mouse kidney (**i**–**k**). MnSOD immunofluorescence (green fluorescence) (**e**–**h**) and its quantification (**l**) in each group. The Tac groups showed mitochondrial disorders such as a lower number, area, and size of mitochondria, and rKlotho administration reversed those changes. Note that the reduction and intensity of MnSOD-positive dots was also improved by rKlotho administration. Scale bars=1 *μ*m (**a**–**d**) or 10 *μ*m (**e**–**h**). The data are presented as the mean±S.E. ^#^*P*<0.05 versus the VH group; ^$^*P*<0.05 versus the rKlotho groups; ^@^*P*<0.05 versus the Tac group

The results shown in Figure 3 implied that rKlotho may regulate mitochondrial function, which is strongly associated with ROS production and apoptosis. Therefore, we studied morphology changes in mouse mitochondria by electron microscopy. Ultrastructural analysis revealed that Tac treatment caused a reduction in the number, area, and size of mitochondria, and rKlotho administration overcame those changes (Figures 3a–d, i–k). We also found that Tac treatment decreased immunoreactivity against MnSOD (green fluorescence), which was mainly localized in the mitochondria of tubular cells. This effect was also significantly inhibited by rKlotho (Figures 3e–h, l).

### Klotho induces MnSOD expression by regulating the PI3K/AKT/FoxO3a pathway during Tac- treatment

**Figure 4:**
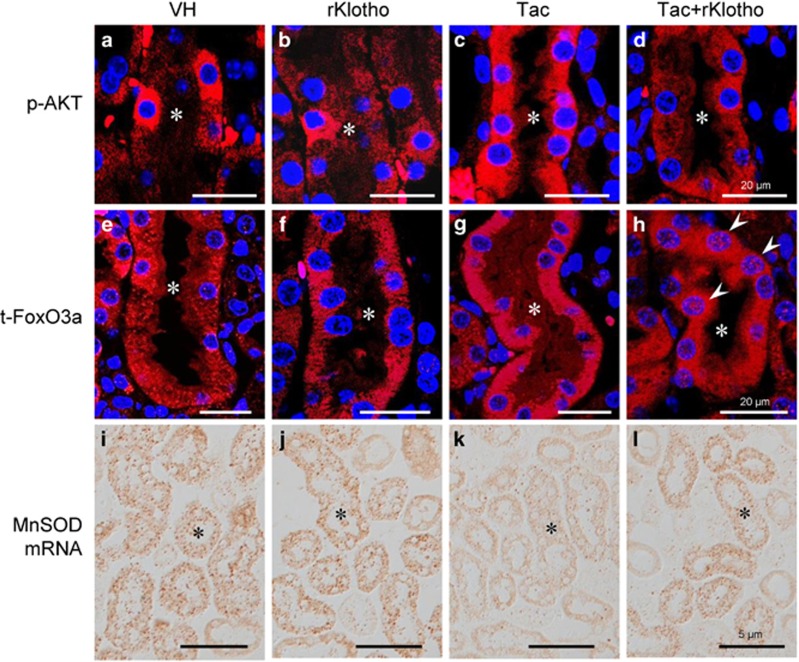
Klotho-induced MnSOD mRNA expression by regulating the PI3K/AKT/FoxO3a pathway in an experimental mouse model. Representative immunofluorescence images of phosphorylated AKT (p-AKT, **a**–**d**) and total FoxO3a (t-FoxO3a, **e**–**h**), and *in situ* hybridization of MnSOD mRNA (**i**–**l**) in tissue sections from mouse kidneys. Arrowheads point to nuclear punctate expression of t-FoxO3a in the Tac+rKlotho group. Asterisks indicate proximal tubules. Scale bars=20 *μ*m (**a**–**h**) or 5 *μ*m (**i**–**l**)

**Figure 5:**
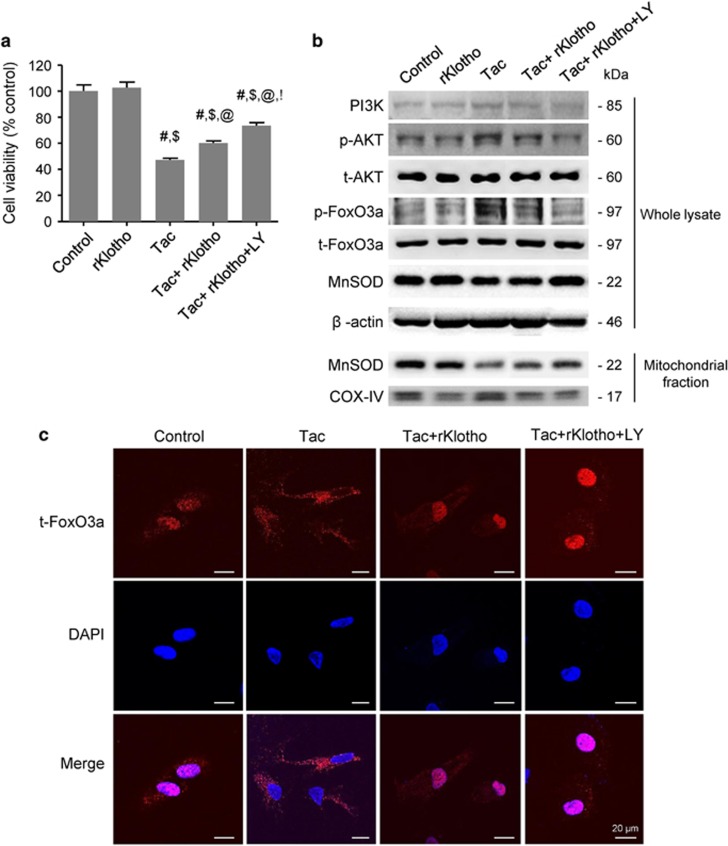
Klotho induces MnSOD expression by regulating the PI3K/AKT/FoxO3a pathway in Tac-treated HK-2 cells. HK-2 cells were seeded in culture plates at 90% confluence. On the next day, the cells were treated with Tac (60 *μ*g/ml) in the absence or presence of 1 *μ*g/ml rKlotho and 25 *μ*M LY294002 (LY, PI3K inhibitor) for 12 h. Before the end of the treatment, CCK-8 solution was added to each well for 2 h to measure the cell viability. (**a**) Cell-viability assays for the experimental group. (**b**) Whole-cell lysates and mitochondrial fractions were collected after each 12- h drug treatment to measure the protein expression of PI3K, phosphorylated AKT (p-AKT), phosphorylated FoxO3a (p-FoxO3a), and MnSOD. All proteins were normalized to *β*-actin or total (t) protein controls (for the whole-cell lysate) and cytochrome c oxidase subunit 4 (COX-IV) (for the mitochondrial fraction). (**c**) After each 12-h drug treatment, the cells were fixed with fixative and then immunofluorescence was performed with an antibody against t-FoxO3a. Nuclear translocation of t-FoxO3a was observed by confocal microscopy. Scale bar=20 *μ*m. The data are presented as the mean±S.E. ^#^*P*<0.05 versus the VH group; ^$^*P*<0.05 versus the rKlotho groups; ^@^*P*<0.05 versus the Tac group; ^!^*P*<0.05 versus the Tac+rKlotho group

To determine whether rKlotho upregulates the MnSOD signaling pathway, we performed immunofluorescence for p-AKT and t-FoxO3a in an experimental mouse kidney section. Confocal microscopy revealed that Tac treatment increased p-AKT expression in proximal tubule, and rKlotho co-treatment reduced this increase (Figures 4a–d). Intense immunofluorescent staining of t-FoxO3a in the cytoplasm was detected in the proximal tubules of the Tac group (Figures 4e–g). Interestingly, t-FoxO3a was also located in the nucleus in the rKlotho co-treated group (Figure 4h). mRNA levels of MnSOD were detected by *in situ* hybridization in tissue sections, and Tac treatment markedly reduced the mRNA level, whereas rKlotho treatment recovered the intensity. Using the HK-2 proximal tubule cell line, we further evaluated whether rKlotho upregulates MnSOD expression via the PI3K/AKT/FoxO3a pathway. We also examined cell viability with or without the PI3K-specific inhibitor LY294002 during Tac and rKlotho treatment in HK-2 cells. The protective effect of rKlotho further increased after LY294002 treatment (Figure 5a). Immunoblot analysis using whole-cell lysates revealed that Tac treatment activated PI3K/AKT, thereby increasing FoxO3a phosphorylation (causing deactivation or cytoplasmic retention) and decreased MnSOD expression (Figure 5b). Similarly, reduction of the MnSOD level by Tac treatment was also increased with rKlotho treatment in protein samples of the mitochondrial fraction.

**Figure 6:**
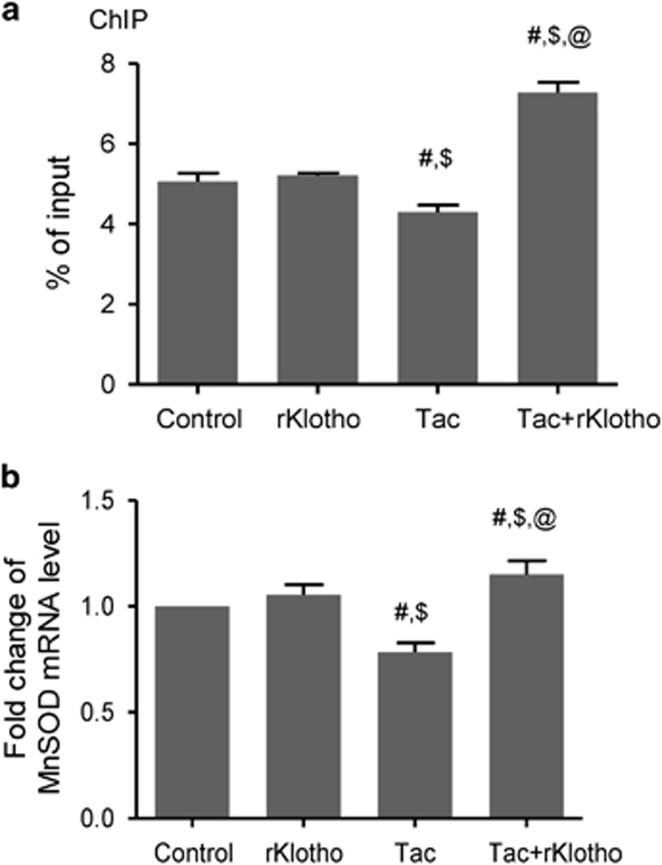
Klotho induces FoxO3a binding to the MnSOD promoter in Tac-treated HK-2 cells. HK-2 cells were seeded in culture plates at 90% confluence. On the next day, the cells were treated with Tac (60 *μ*g/ml) in the absence or presence of 1 *μ*g/ml rKlotho. After 12 h, the cells were harvested to detect MnSOD transcription activity after nuclear FoxO3a translocation using a ChIP assay and quantitative real-time PCR (q-PCR) for MnSOD expression. (**a**) The FoxO3a protein/MnSOD promoter complex was analyzed by q-PCR with primer pairs for the MnSOD promoter region containing a FoxO3a-binding element. (**b**) MnSOD mRNA was detected by q-PCR in whole-cell lysates. Relative MnSOD expression was presented after normalization to cyclophilin A expression. The data are presented as the mean±S.E. ^#^*P*<0.05 versus the control group; ^$^*P*<0.05 versus the rKlotho groups; ^@^*P*<0.05 versus the Tac group

Next, we confirmed that Klotho-induced FoxO3a nuclear translocalization by immunostaining for total FoxO3a (t-FoxO3a) expression. t-FoxO3a mainly localized to the cytoplasm in the Tac group, but nuclear t-FoxO3a immunoreactivity occurred after Klotho treatment. (Figure 5c). We studied t-FoxO3a immunoreactivity in the LY294002-treated group as a positive control doe nuclear staining. Furthermore, a chromatin immunoprecipitation (ChIP) assay revealed that rKlotho treatment increased FoxO3a binding to the MnSOD promoter and the MnSOD mRNA level (Figures 6a and b).

### Klotho reduces Tac-induced mitochondrial ROS production and mitochondrial dysfunction

**Figure 7:**
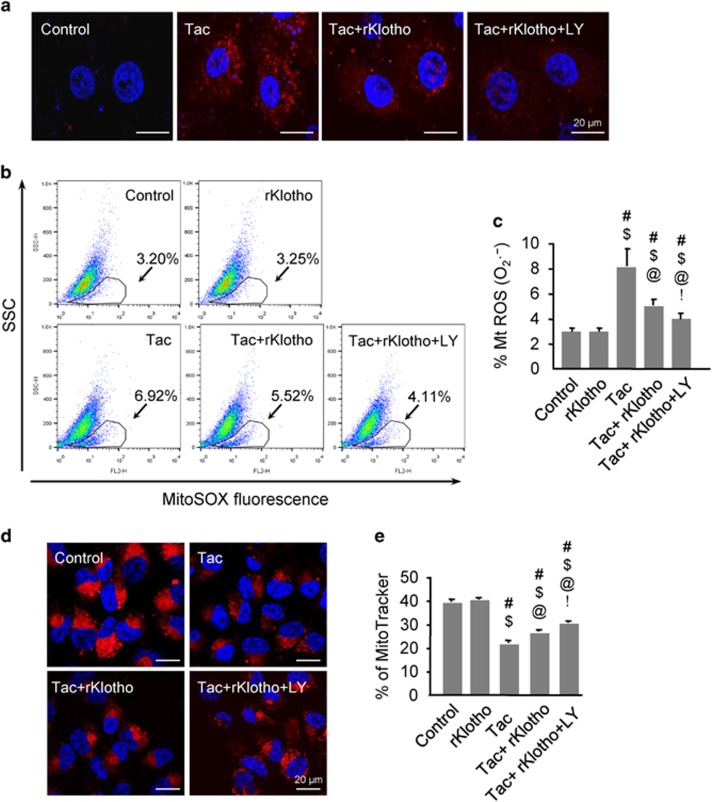
Klotho reduces Tac-induced mitochondrial ROS production and mitochondrial damage in HK-2 cells. HK-2 cells were seeded in culture plate at 90% confluence. On the next day, the cells were treated with Tac (60 *μ*g/ml) in the absence or presence of 1 *μ*g/ml rKlotho and 25 *μ*M LY294002 (LY, PI3K inhibitor) for 12 h. The cells were exposed in MitoSOX or MitoTracker, then analyzed by flow cytometry and confocal microscopy. MitoSOX staining to detect mitochondrial superoxide anion (O2^−^) levels by confocal microscopy (**a**) or flow cytometric analysis (**b** and **c**). MitoTracker staining to detect the number of mitochondria by confocal microscopy (**d**). (**e**) Relative fluorescence intensity of MitoTracker staining by flow cytometry. LY; LY294002. Scale bar=20 *μ*m. The data are presented as the mean±S.E. ^#^*P*<0.05 versus the VH group; ^$^*P*<0.05 versus the rKlotho groups; ^@^*P*<0.05 versus the Tac group; ^!^*P*<0.05 versus the Tac+rKlotho group

**Figure 8:**
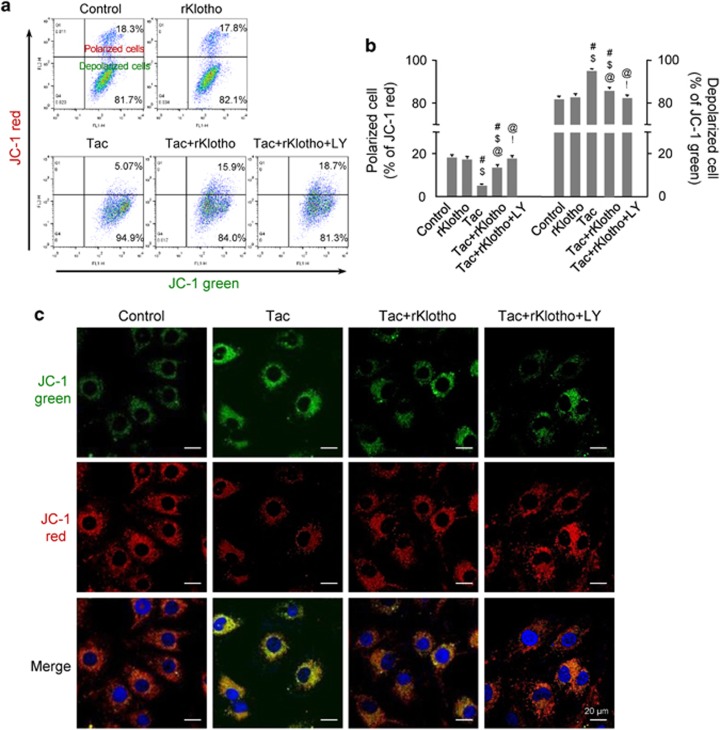
Klotho improves the mitochondrial membrane potential in Tac-treated HK-2 cells. HK-2 cells were seeded in culture plates at 90% confluence. On the next day, the cells were treated with Tac (60 *μ*g/ml) in the absence or presence of 1 *μ*g/ml rKlotho and 25 *μ*M LY294002 (LY, PI3K inhibitor) for 12 h. The cells were labeled with JC-1 to evaluate the mitochondrial membrane potential (ΔΨm) and then analyzed by flow cytometry and confocal microscopy. (**a** and **b**) Flow cytometry plots and a quantitative graph for JC-1 labeling. (**c**) Confocal microscopy images for JC-1 staining. LY; LY294002. Scale bar=20 *μ*m. The data are presented as the mean±S.E. ^#^*P*<0.05 versus the VH group; ^$^*P*<0.05 versus the rKlotho groups; ^@^*P*<0.05 versus the Tac group; ^!^*P*<0.05 versus the Tac+rKlotho group

**Figure 9:**
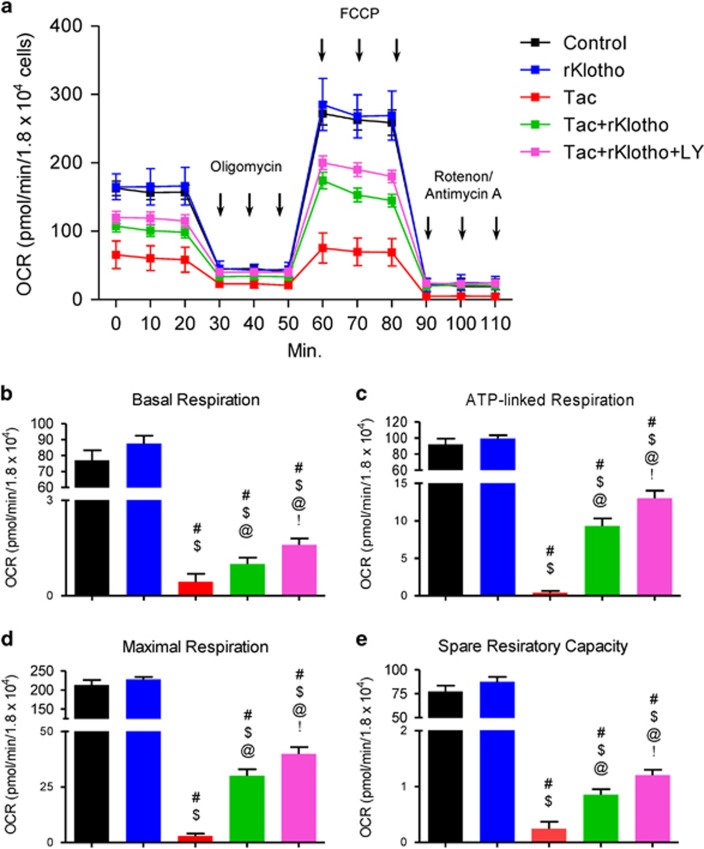
Klotho reduces Tac-induced mitochondrial dysfunction in HK-2 cells. HK-2 cells were seeded in culture plates at 90% confluence. On the next day, the cells were treated with Tac (60 *μ*g/ml) in the absence or presence of 1 *μ*g/ml rKlotho and 25 *μ*M LY294002 (LY, PI3K inhibitor). After a 12-h treatment, the cells were incubated in a non-CO_2_ incubator for 1 h. Next, the ATP synthase inhibitor oligomycin, the uncoupler FCCP, or the respiratory chain complex I and III inhibitor rotenone/antimycin A were added to the culture medium, as indicated (**a**). The areas under the curve for basal respiration (**b**), ATP production (**c**), maximal respiration (**d**), and spare respiratory capacity (**e**) were calculated from the OCR. The data are presented as the mean±S.E. ^#^*P*<0.05 versus the VH group; ^$^*P*<0.05 versus the rKlotho groups; ^@^*P*<0.05 versus the Tac group; ^!^*P*<0.05 versus the Tac+rKlotho group

Next, we evaluated whether Klotho-dependent MnSOD induction via the PI3K/AKT/FoxO3a pathway regulation decreased Tac-induced mitochondrial ROS accumulation. MitoSOX Red staining for mitochondrial superoxide (O2^−^) along with confocal microscopy and flow cytometric analysis in HK-2 cells revealed that rKlotho significantly decreased Tac-induced MitoSOX Red fluorescence, and suppression of PI3K by LY294002 further reduced its expression (Figures 7a–c). We also found that MitoTracker fluorescence significantly decreased in the Tac group and the addition of rKlotho significantly the fluorescence, demonstrating that rKlotho reduced mitochondrial damage (Figures 7d and e). In addition, the significantly decreased number of polarized cells (JC-1 red) in the Tac group was reduced by rKlotho treatment. In contrast, the increase in depolarized cells was reduced in the Tac+rKlotho group (Figure 8). The oxygen-consumption rate (OCR) of cells was assessed, as shown in Figure 9. rKlotho-treated cells showed a higher OCR than cells treated with Tac alone (Figure 9a). Compared with Tac alone-treated cells, Tac+rKlotho-treated cells showed significantly higher basal respiration, ATP-linked respiration, maximal respiration, and spare respiratory capacity. These data suggested that rKlotho may protect against Tac-induced mitochondrial dysfunction (Figures 9b–e). Blocking of PI3K by LY294002 during Tac+rKlotho treatment further increased mitochondrial function.

### Klotho reduces Tac-induced apoptosis in HK-2 cells

**Figure 10:**
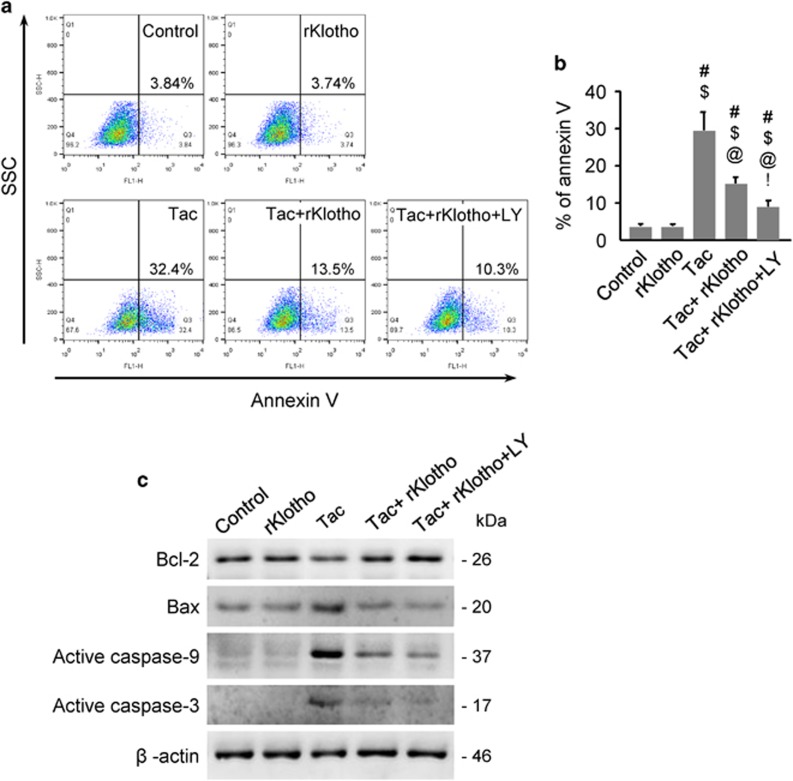
Klotho reduces Tac-induced apoptosis in HK-2 cells. HK-2 cells were seeded in culture plates at 90% confluence. On next day, the cells were treated with Tac (60 *μ*g/ml) in the absence or presence of 1 *μ*g/ml rKlotho and 25 *μ*M LY294002 (LY, PI3K inhibitor) for 12 h. Before the end of the treatment, annexin V solution was added to each well for 15 min to measure apoptosis, which was analyzed by flow cytometry. Whole-cell lysates were collected after each 12-h drug treatment to measure the relative protein expression levels of Bcl-2, Bax, active caspase-9 and active caspase-3. (**a** and **b**) Flow cytometry histograms and a graph of annexin V labeling. (**c**) Representative western blots for Bcl-2, Bax, active caspase-9, and active caspase-3. All proteins are normalized to *β*-actin expression. LY; LY294002. The data are presented as the mean±S.E. ^#^*P*<0.05 versus the VH group; ^$^*P*<0.05 versus the rKlotho groups; ^@^*P*<0.05 versus the Tac group; ^!^*P*<0.05 versus the Tac+rKlotho group

To examine whether rKlotho inhibits mitochondrial pathways associated with apoptosis, we performed flow cytometric analysis by annexin V staining and immunoblotting against pro- or anti-apoptotic genes. The higher percent of annexin V-positive cells after Tac treatment was significantly reduced by rKlotho treatment and even more so by LY294002 (Figures 10a and b). Using whole-cell lysates, we found that Tac-dependent Bcl-2 downregulation was reversed by rKlotho. Pro-apoptotic markers (Bax, activated caspase-9 and -3) were markedly increased by Tac treatment, and this phenomenon was blocked in rKlotho-treated cells. PI3K inhibition with LY294002 further improved the anti-apoptotic effect of rKlotho (Figure 10c).

## Discussion

The results of present study clearly demonstrated that Klotho inhibited PI3K/AKT-mediated phosphorylation of FoxO3a and subsequent enhancement of FoxO3a binding to the MnSOD promoter. Indeed, Klotho increased MnSOD mRNA and protein expression in mitochondria during Tac-induced toxicity in HK-2 cells. These findings suggest that Klotho increases resistance to Tac-induced oxidative stress in the kidneys via negative regulation of the PI3K/AKT pathway and that FoxO3a-mediated MnSOD expression is involved in this process.

We previously demonstrated that Tac-induced nephropathy caused downregulation of renal Klotho, and drugs with antioxidant potential such as statin, angiotensin II blockade, and N-acetylcysteine,^25, 26, 27, 28^ increased Klotho expression. This finding suggests a close association between Klotho and oxidative stress in Tac-induced renal injury, which was confirmed by showing that Klotho +/− mice showed more Tac-induced renal injury than wild-type littermates.^29^ In this study, we determined whether exogenous treatment of Klotho protein protects against Tac-induced oxidative injury. Co-treatment with the Klotho protein in mice improved renal function and attenuated tubulointerstitial fibrosis and renal KIM-1 expression, compared with Tac treatment alone. Next, we evaluated whether Klotho treatment decreases Tac-induced oxidative stress and apoptosis, and found that Klotho treatment dramatically downregulated oxidative stress markers (8-OHdG and 4-HHE) and reduced the number of TUNEL-positive cells. These findings suggest that Klotho treatment may be beneficial in reducing structural and functional kidney impairment during Tac treatment.

We focused on mitochondrial ROS and MnSOD, based on previous reports that mitochondria are the primary cellular source of ROS and that MnSOD is as an important antioxidant enzyme in the mitochondria.^30^ Regarding the upregulation of MnSOD by Klotho, multiple mechanisms may contribute to protein kinase C-, PI3K/AKT-, and MAP kinase-mediated pathways. In the present study, we explored whether Klotho influences the PI3K/AKT pathway because downstream targets of the PI3K/AKT signaling pathway include those resulting from FoxO3a-mediated MnSOD expression.^5, 31^ In kidney tissue sections, Klotho co-treatment reduced detection of phosphorylated AKT and enhanced nuclear FoxO3a expression. Reduced mRNA and protein levels of MnSOD (evaluated by *in situ* hybridization and immunofluorescence, respectively) were also observed following Klotho co-treatment (Figure 4). This finding suggests an association between the PI3K/AKT/FoxO-signaling pathway and MnSOD, and these relations might contribute to the anti-oxidative properties of Klotho against Tac-induced oxidative injury.

Next, we studied causal relationships between the PI3K/AKT pathway and Klotho activity on *in vitro* study. *In vitro* study showed that co-treatment with rKlotho and Tac promoted nuclear FoxO3a translocation. Negative regulation of PI3K/AKT was confirmed with by treatment of with the LY294002, PI3K inhibitor. ChIP assays revealed that Klotho increased FoxO3a binding to the native MnSOD gene promoter, which was associated with increased MnSOD mRNA and protein expression. These results demonstrated that Klotho inhibited the Tac-induced PI3K/AKT pathway, thereby promoting translocation to the nucleus, which occurs is stabilized via direct binding of FoxO3a to the MnSOD promoter.

Next, we examined whether Klotho-induced MnSOD expression in mitochondria helped improved Tac-induced mitochondrial dysfunction mediated by inhibiting the PI3K/AKT pathway. Mitochondria function (assessed by the OCR) showed that Klotho-treated cells had significantly higher basal respiration, ATP-linked respiration, maximal respiration, and spare respiratory capacity. In addition, mitochondrial membrane depolarization and excessive mitochondrial ROS formation during Tac treatment were also recovered by Klotho treatment. These protective effects of Klotho in mitochondria were related with to PI3K/AKT pathway by blocking PI3K with LY294002. In addition, we found that mitochondrial-associated apoptosis during Tac treatment was decreased by Klotho treatment via Bcl-2 upregulation. These data suggested that the addition of Klotho protects against Tac-induced mitochondria dysfunction and apoptosis by negatively regulating the PI3k/AKT pathway.

Our study had several limitations. First, it remains unclear whether the ability of Klotho to confer oxidative stress resistance is entirely dependent on MnSOD induction, although this activity may be crucial for the anti-oxidative properties of Klotho. Overexpression of catalase removed ROS (catalase) in mitochondria and extended the lifespan of mice.^32, 33, 34^ A peroxiredoxin/thioredoxin-associated mechanism was associated with Klotho-induced ROS removal following brain injury.^35^ Second, we cannot explain clearly why Klotho treatment did not reverse weight loss in Tac-treated mice. However, we speculate that the protective effects of Klotho were not enough to promote weight recovery under these experimental conditions. Additional experiments are needed to resolve this problem in further investigations.

**Figure 11:**
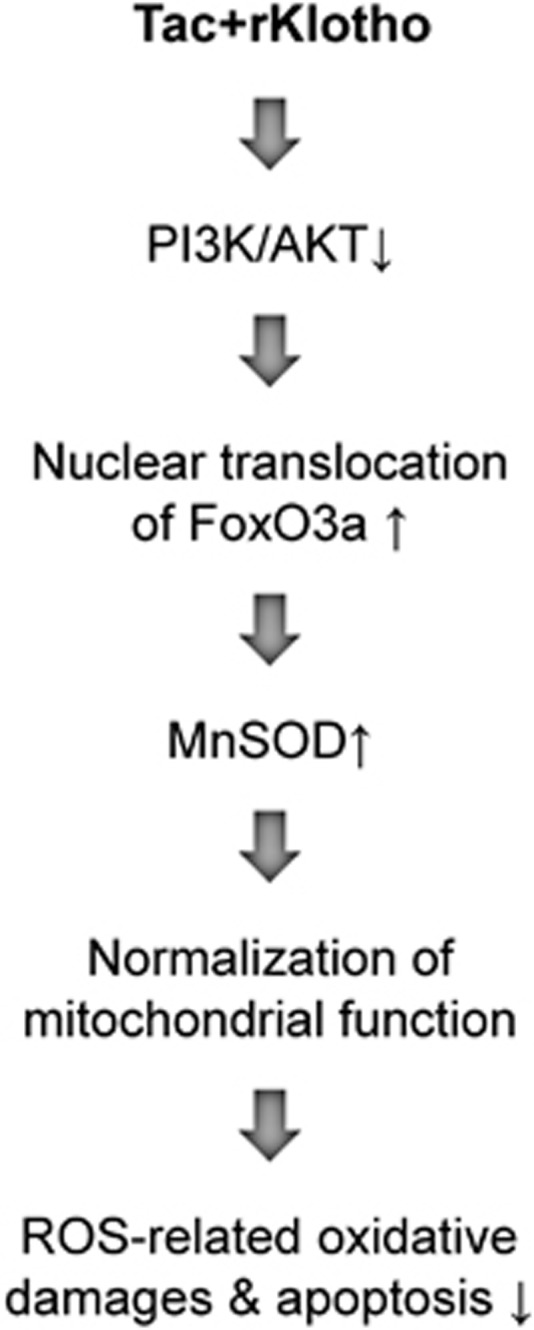
Proposed mechanism of the protective effect of Klotho during Tac-induced renal injury

The proposed mechanism underlying the protective effect of Klotho in Tac-induced renal injury is summarized in Figure 11. Tac regulates PI3K/AKT-mediated phosphorylation of FoxO3a, which suggests that FoxO3a may be retained in the cytoplasm in an inactive form. However, Klotho promoted the nuclear export of FoxO3a by inhibiting PI3K/AKT activity and increasing MnSOD mRNA and protein expression. Through this mechanism, Klotho may protect against Tac-induced oxidative damage and apoptotic cell death. Finally, our results suggested that the Klotho protein or Klotho-enhancing compounds may provide treatment options for nephrotoxicity in the future.

## Materials and methods

### Ethics statement

All procedures were performed in strict accordance with the recommendations of the ethical guidelines for animal studies. All experimental animal care protocols were approved by the Animal Care and Use Committee of the Catholic University of Korea (CUMC-2013-0056-02). Animals were killed under xylazine/rompun anesthesia, and every effort was made to minimize animal suffering.

### Tac-induced renal injury mouse model

Eight-week-old male BALB/c mice (Orient Bio, Seongnam-Si, Korea) were housed with a 12-h/12-h light/dark cycle, a 0.01% salt diet (Research Diets, New Brunswick, NJ, USA), and water *ad libitum*. After acclimation for 1 week, weight-matched mice were randomized to four groups (*n*=8/group) and treated together subcutaneously with 1.5 mg/(kg day) Tac (Prograft; Astellas Pharma, Ibaraki, Japan) or 10 ml/(kg day) VH (olive oil; Sigma-Aldrich, St. Louis, MO, USA), with or without recombinant mouse Klotho (rKlotho; 10 *μ*g/kg once every 2 days, intraperitoneal injection; R&D System, Minneapolis, MN, USA) for 4 weeks. The rKlotho used was the shed, circulating form of Klotho, not the transmembrane or secreted form. Administration routes and drug doses were chosen based on previous studies.^2, 29^ After the 4-week treatment, animals were housed individually in metabolic cages (Tecniplast, Buguggiate, Italy) to measure urine volumes over 24 h. Animals were then anaesthetized, and blood samples and tissue specimens were obtained for further analysis.

### Creatinine measurements

Scr was measured using a quantitative enzyme colorimetric method (Stanbio Laboratory, Boerne, TX, USA), according to the manufacturer’s instructions.

### Renal fibrosis

Renal histological assessment was defined as the development of tubulointerstitial fibrosis using Trichrome-stained tissue sections.^36^ The extent of fibrosis was estimated in minimum of 20 fields per section by counting the percentage of injured area per field using the polygon program (TDI Scope Eye Version 3.6 for Windows; Seoul, Korea). Histopathological analysis was performed in randomly selected cortical fields of sections by a pathologist blinded to the identity of the treatment groups.

### Antibodies

The following primary antibodies were used for immunoblot analysis or immunohistochemical staining: anti-KIM-1 (ab56015; Abcam, Cambridge, UK), anti-8-OHdG (MOG-100 P; JaICA, Shizuoka, Japan), anti-4-HHE (MHH-030n; JaICA), anti-MnSOD (ab16953; Abcam), anti-PI3K (610045; BD Transduction Laboratories, San Jose, CA, USA), anti-p-AKT (Ser473) (9271 S; Cell Signaling Technology, Danvers, MA, USA), anti-t-AKT (9272 S; Cell Signaling Technology), anti-p-FoxO3a (9466 S; Cell Signaling Technology), anti-t-FoxO3a (2497 S; Cell Signaling Technology), anti-*β*-actin (A5441; Sigma-Aldrich), anti-COX-IV (A301-899 A; Bethyl Laboratories, Montgomery, TX, USA), anti-Bcl-2 (sc-492; Santa Cruz Biotechnology, Santa Cruz, CA, USA), anti-Bax (DB005; Delta Biolabs, Gilroy, CA, USA), anti-active caspase-9 (9505 S; Cell Signaling Technology), and anti-active caspase-3 (AB3623; Millipore Corporation, St. Charles, MO, USA).

### Immunohistochemical staining

Dewaxed sections were incubated in retrieval solution (pH 6.0), methanolic H2O2, and 0.5% Triton X-100 and then washed in phosphate-buffered saline. Nonspecific binding sites were blocked in 10% normal donkey serum (Jackson ImmunoResearch, West Grove, PA, USA). Sections were incubated overnight at 4 °C with primary antibodies and then with peroxidase- or Alexa Fluor 488 or Cyanine 3-conjugated secondary antibodies (Molecular Probes, Carlsbad, CA, USA, Jackson ImmunoResearch) for 2 h at room temperature (RT). Peroxidase activity was detected using 3,3’-diaminobenzidine (DAB; Vector Laboratories, Burlingame, CA, USA) as a chromogen. For confocal images, the tissue sections were stained with 4’,6-diamidino-2-phenylindole (Vector Laboratories). Stained tissues were viewed using a Zeiss LSM700 confocal microscope (Carl Zeiss Microscopy, GmbH, Jena, Germany). Quantification was performed inof ~20 randomly selected areas was performed per for each animal in each group. Quantitative analysis was performed by calculating the percent positive area showing the same intensity, using histogram equalization (TDI Scope Eye).

### TUNEL staining

Apoptotic cells in tissue sections were detected by the TUNEL method and staining with the *In Situ* Apoptosis Detection Kit (Millipore Corp.). TUNEL-positive cells were counted in ~20 randomly selected non-overlapping areas per animal in each group.

### *In situ* hybridization

Visualization of MnSOD mRNA transcripts in tissues was done according to the supplier instructions for the RNAScope 2.5 HD Detection Kit (322371; Advanced Cell Diagnostics, Hayward, CA, USA) using a validated probe for mouse MnSOD (439361; Advanced Cell Diagnostics). In brief, 5 *μ*m slide-mounted sections were heated for 60 min at 60 °C in a HybEZTM hybridization oven (Advanced Cell Diagnostics). Tissues were dewaxed in xylene followed by dehydration in an ethanol series and air dried for 5 min. Tissue sections were incubated with pretreatment 1 solution (endogenous peroxidase block) for 10 min at RT. Slides were rinsed by immersion in double-distilled water (ddH2O), followed by immersion in pretreatment 2 solution (antigen-retrieval citrate buffer) for 15 min at 100–104 ºC. Slides were washed in ddH2O and pretreatment 3 (protease) was applied for 30 min at 40 ºC. Slides were washed in ddH2O, and target or control probes were incubated at 40 °C for 2 h followed by rinsing in wash buffer (Advanced Cell Diagnostics) for 2 min at RT. Signal-amplification reagents 1–6 were applied sequentially for 30 min, 15 min, 30 min, 15 min, 30 min, and 15 min, respectively. Slides were rinsed in wash buffer for 2 min between amplification reagents. Incubations with amplifier reagents 1 to 4 were performed at 40 ºC, whereas incubations with amplifier reagents 5 and 6 were performed at RT. Positive signals were visualized using DAB (Vector Laboratories). After drying for 15 min at 60 ºC, slides were coverslipped using mounting media (Fisher Scientific, Medford, MA, USA). The positive control probe consisted of a proprietary probe for Bos taurus cyclophilin B, whereas the negative control probe targeted dapB of *Bacillus subtilis*.

### Detection of 8-OHdG in urine

The end product of oxidative DNA damage was evaluated based on the level of the DNA adduct 8-OHdG in 24-h urine. The 8-OHdG concentration in urine was measured using a competitive ELISA (Cell Biolabs, San Diego, CA, USA).

### Electron microscopy

After fixation in 2.5% glutaraldehyde in 0.1 M phosphate buffer, renal cortex tissues were post-fixed with 1% O_S_O_4_ and embedded in Epon 812. Ultrathin sections were cut, stained with uranyl acetate/lead citrate, and photographed with a JEM-1200EX transmission electron microscope (JEOL Ltd., Tokyo, Japan). Sections were scanned randomly at 20 different spots per sample at × 5000 magnification. The numbers, areas, and sizes of mitochondria were measured in 20 random proximal tubular cells, using imaging software (TDI Scope Eye).

### Cell culture

HK-2 cells from an immortalized human proximal tubular epithelial cell line were grown in Dulbecco’s modified Eagle’s medium (DMEM) containing 10% fetal bovine serum supplemented with 100 U/ml penicillin and 100 *μ*g/ml streptomycin, and incubated at 37 °C in a humidified atmosphere containing 5% CO_2_. The cells were seeded in culture plates and treated with Tac (60 *μ*g/ml) and recombinant human Klotho (rKlotho; 1 *μ*g/ml, R&D System), with or without LY294002 (25 *μ*M, Sigma-Aldrich) for 12 h.

### Cell-viability assay

Cells were seeded in 96-well plates at a density of 2 × 10^4^ cells/well for 24 h and then subjected to various treatments for the specified periods. Before the end of the treatments, Cell Counting Kit (CCK)-8 solution (Dojindo Molecular Technologies, Kumamoto, Japan) was added to each well for 2 h. Absorbance was measured at 450 nm using a VersaMax ELISA Reader (Molecular Devices, Sunnyvale, CA, USA).

### Immunoblot analysis

Whole cells were lysed in PRO-PREP protein-extraction solution (Intron Biotechnology, Seongnam-si, Korea) according to the manufacturer’s instructions. Equal amounts of protein were subjected to immunoblotting analysis with primary antibodies. Signals were detected using an enhanced chemiluminescence system (ATTO Corp., Tokyo, Japan). Quantification of relative densities was performed with the control group set at 100% densities were normalized to that of *β*-actin bands from the same gel (Quantity One version 4.4.0; Bio-Rad, Hercules, CA, USA).

### Mitochondrial fractionation

Cells were harvested using a scraper and centrifuged at 500 × *g*. The cell pellet was washed by resuspension in phosphate-buffered saline. Mitochondria were separated by using the Mitochondria Isolation Kit (Pierce Biotechnology, Rockford, IL, USA). Mitochondria fractions were confirmed using a COX-IV antibody (A301-899A; Bethyl Laboratories).

### ChIP experiments

ChIP was performed using a commercial kit (Millipore Corporation). In brief, cells were incubated with or without the rKlotho protein for 12 h during Tac treatment, after which they were cross-linked with 1% formaldehyde. After washing, the cells were sonicated and immunoprecipitated with an anti-FoxO3a antibody (ab12162; Abcam) overnight at 4 ºC. After elution and reverse cross-linking the antibody/DNA complexes, the cellular DNA was purified using a DNA purification kit (Qiagen, Hilden, Germany) and analyzed by quantitative real-time PCR (q-PCR) using primer pairs covering the MnSOD promoter region containing a FoxO3a-binding element at position -1249 (5′-GAGTATCTATAACCTGGTCCCAGCC-3′ and 5′-GCTGAACCGTTTCCGTTGCTTCTTGC-3′). Data were shown as the amount of DNA relative to input.

### qRT-PCR

Total RNA from cultured cells were isolated using RNA-spin Total RNA Extraction Kit (Intron Biotechnology, Seongnam-si, Gyeonggi-do). First-strand cDNA was synthesized and subjected to q-PCR using SYBR Green Master Mix in a LightCycler 480 system (Roche, Rotkreuz, Switzerland). Gene expression was normalized to cyclophilin A expression using the change-in-threshold method and primers with the following sequences: 5′-GGTCCCAAAGACAGCAGAAA-3′ and 5′-GTCACCACCCTGACACATAAA-3′.

### Mitochondrial ROS detection

After treatment with the drug or VH, mitochondrial ROS (superoxide anion) were detected using MitoSox Red (Invitrogen, Carlsbad, CA, USA) for 30 min at 37 °C according to the manufacturer’s instructions and analyzed using a FACS Calibur flow cytometer (BD Biosciences, San Jose, CA, USA) or an LSM700 confocal microscope (Carl Zeiss Microscopy GmbH). Forward and side scatter data were collected (10 000 events per sample).

### Determination of mitochondrial damage

Mitochondrial integrity was assessed using MitoTracker Deep Red FM (Invitrogen). After treatment with the drug or VH for 20 min at 37 °C per the manufacturer’s instructions, cells were analyzed using a FACS Calibur flow cytometer or an LSM700 confocal microscope.

### Mitochondrial membrane potential (ΔΨm)

The ΔΨm of HK-2 cells was measured using the fluorescent, lipophilic cationic probe, JC-1 (Cayman, Ann Arbor, MI, USA). When live cells are incubated with JC-1, the dye penetrates the plasma membrane of cells as monomers and JC-1 uptake into mitochondria is driven by the ΔΨm. Functional mitochondria are polarized and JC-1 is rapidly taken up. This uptake raises the JC-1 concentration leading to the formation of aggregates (J-aggregates) within mitochondria. Excited by a 488 nm laser, J-aggregates provoke a red spectral shift emission (590 nm). JC-1 does not accumulate in depolarized mitochondria, but instead remains in the cytoplasm as monomers, which emit green fluorescence (525 nm). Thus, in healthy cells, JC-1 fluorescence is seen in both the green (FL-1) and red (FL-2) channels, and a loss of red fluorescence indicates depolarized mitochondria. After treatment with the drug or VH, the cells were incubated with JC-1 staining solution for 20 min at 37 °C according to the manufacturer’s instructions and then analyzed using a FACS Calibur flow cytometer or an LSM700 confocal microscope.

### OCR experiments

The cellular OCR rate was assessed in real-time with an XF24 Extracellular Flux Analyzer (Seahorse Biosciences, Billerica, MA, USA). After treatment with the drug or VH, the cell medium was changed to running medium (DMEM supplemented with 5.5 mM glucose, 1 mM sodium pyruvate, 4 mM L-glutamine, pH 7.4) and incubated at 37 °C in a non-CO_2_ incubator for 1 h. The mitochondrial inhibitors used were the ATP synthase inhibitor oligomycin (1 *μ*M), carbonyl cyanide-4 (trifluoromethoxy) phenylhydrazone (FCCP; 0.5 *μ*M), and the complex I and III inhibitor rotenone/antimycin A (0.5 *μ*M). OCR values were then normalized to the protein content of each sample. Mitochondrial function parameters were determined using these mitochondrial inhibitor compounds as modulators to determine several bioenergetics and mitochondrial function parameters, including basal respiration, ATP production, maximal respiration, and spare respiratory capacity, as described by Dott *et al.*^37^ Three to four wells were used for each experimental group.

### Apoptosis

After treatment with the drug or VH, trypsinized cells were incubated with 5 *μ*l of fluorescein isothiocyanate-conjugated annexin V (BD Biosciences) in 1 × binding buffer (BD Biosciences) for 15 min at RT, according to the manufacturer’s protocol. The stained cells were analyzed by flow cytometry on a FACS Calibur instrument. Values are expressed as the percentage of fluorescent cells relative to the total cell count.

### Statistical analysis

The data are expressed as the mean±standard error (S.E.) of at least three independent experiments. Multiple comparisons between groups were performed by one-way analysis of variance with Bonferroni’s *post hoc* test, using SPSS software (Version 19.0; IBM Corp., Armonk, NY, USA). Results with *P*-values<0.05 were considered significant.

## Figures and Tables

**Table 1:** Effect of administration of rKlotho on Tac-induced renal injury in mice

	**VH**	**rKlotho**	**Tac**	**Tac+rKlotho**
Body weight (g)	31±1	32±1	27±1^1^	25±1
Water intake (ml/day)	2.2±0.14	2.3±0.19	4.5±0.67^1,2^	2.9±0.33^3^
Urine volume (ml/day)	0.3±0.03	0.4±0.02	0.8±0.11^1,2^	0.33±0.10^3^
Scr (mg/dl)	0.15±0.2	0.15±0.2	0.4±0.3^1,2^	0.2±0.2^3^
KIM-1 (%)	10.9±2.0	9.0±3.4	19.9±4.0^1,2^	7.6±3.3^3^

Abbreviations: KIM-1, kidney injury marker-1; rKlotho, recombinant Klotho; Scr, serum creatinine; Tac, tacrolimus; VH, vehicle

The values shown are the mean±S.E. (*n*=8) ^1^P<0.05 *versus* VH; ^2^P<0.05 *versus* rKlotho; ^3^P<0.05 *versus* Tac.
